# Supplementary material for: Two-Channel Indirect-Gap Photoluminescence and Competition between the Conduction Band Valleys in Few-Layer MoS2
Source: Nanomaterials (Basel). 2023 Dec 30;14(1):96. doi: 10.3390/nano14010096 (PMC10780461; doi:10.3390/nano14010096)
Supplement: Supplementary file 1 [file nanomaterials-14-00096-s001.zip › nanomaterials-2748032-supplementary.pdf]

Supplementary Materials

Two-Channel Indirect-Gap Photoluminescence and Competition between the Conduction Band Valleys in Few-Layer MoS_2

Ayaz H. Bayramov [1], Elnur A. Bagiyev [1], Elvin H. Alizade [1], Javid N. Jalilli [1], Nazim T. Mamedov [1,2*], Zakir A. Jahangirli [1,2], Saida G. Asadullayeva [1], Yegana N. Aliyeva [2], Massimo Cuscunà [3,*], Daniela Lorenzo [3], Marco Esposito [3], Gianluca Balestra [3,4], Daniela Simeone [3], David Maria Tobaldi [3], Daniel Abou-Ras [5], and Susan Schorr [5,6]

[1] Institute of Physics, Ministry of Science and Education, Baku Az1143, Azerbaijan; bayramov@physics.science.az (A.H.B.); elnur.bagiyev@physics.science.az (E.A.B.); elvin.alizada@physics.science.az (E.H.A.); j.jalilli@physics.science.az (J.N.J.); zakirchangirli@physics.science.az (Z.A.J.); s.asadullayeva@physics.science.az (S.G.A.)
[2] Institute of Physical Problems, Baku State University, Ministry of Science and Education, Baku Az1148, Azerbaijan; yeganaaliyeva.n@bsu.edu.az (Y.N.A.)
[3] National Research Council, Institute of Nanotechnology (NANOTEC), University c/o Campus Ecotekne, Via per Monteroni, 73100 Lecce, Italy; daniela.lorenzo@nanotec.cnr.it (D.L.); marco.esposito@nanotec.cnr.it (M.E.); gianluca.balestra@nanotec.cnr.it (G.B.); daniela.simeone@nanotec.cnr.it (D.S.); david.tobaldi@nanotec.cnr.it (D.M.T.)
[4] Department of Mathematics and Physics "Ennio De Giorgi", University of Salento, c/o Campus Ecotekne, Via per Monteroni, 73100 Lecce, Italy;
[5] Helmholtz-Zentrum Berlin for Materials and Energy (HZB), Department of Structure and Dynamics of Energy Materials, 14109 Berlin, Germany; daniel.abou-ras@helmholtz-berlin.de (D.A.-R.); susan.schorr@helmholtz-berlin.de (S.S.)
[6] Institute of Geological Sciences, Free University of Berlin, 14195 Berlin, Germany

*Correspondence: n.mamedov.physics@bsu.edu.az (N.T.M.); massimo.cuscuna@cnr.it (M.C.)

S1

Depending on the deposition temperature and other technological parameters, the MoO_3 thin films prepared by plasma enhanced atomic layer deposition were either crystalline (c) or amorphous (a), as shown in Fig. S1a. As found from the optical studies using spectroscopic ellipsometry, the photon energy dependence of absorption coefficient α, retrieved from the dielectric function (DF) of c-MoO_3 noticeably differ from that of a-MoO_3 (Fig. S1b). Vertical black dashed line in Fig. S1b indicates the value (3.01 eV) of band gap energy of c-MoO_3. Two vertical red dashed lines indicate the value (2.84 eV) of band gap and the value (2.65 eV) of the energy gap in a-MoO_3 without contribution of Urbach energy (E_u) [44], respectively.

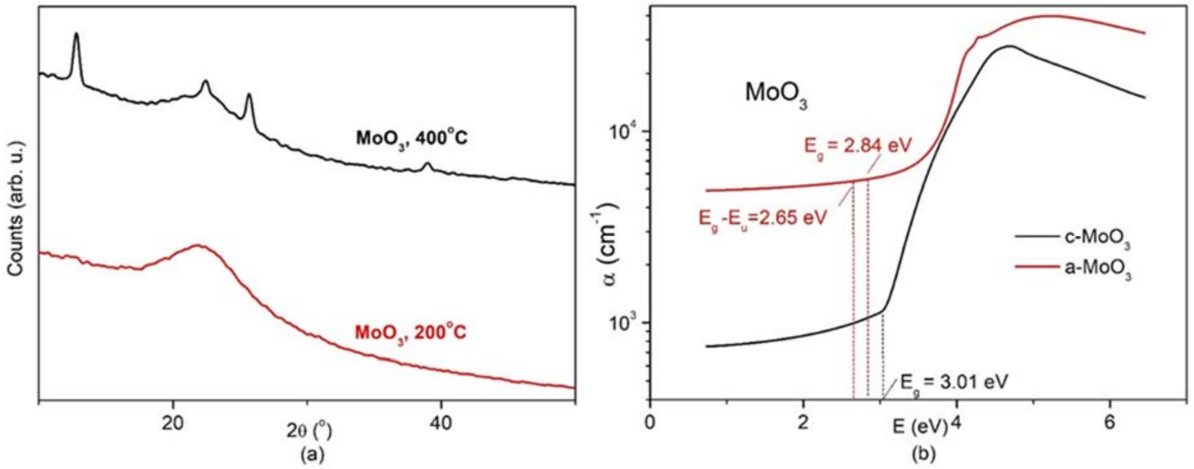

Figure S1. (a): Typical XRD patterns of thin films with crystalline c-MoO_3 (black line) and amorphous a-MoO_3, obtained at different deposition temperatures; (b): Absorption coefficient of c-MoO_3 (black line) and a-MoO_3 (red line).

It is important to mention that the α and DF of the c-MoO_3 films that were further subjected to sulfurization had not shown a broad absorption band around 1.4 eV, which is a distinct feature of c-MoO_3 with oxygen deficiency that creates mid-gap states in c-MoO_3 [45, 46]. In other words, sulfurization of MoO_3 was performed on the films that were free from the frequently encountered defects associated with oxygen deficiency.

S2

Raman spectra of the studied $MoS_2/SiO_2/Si$ films with 3 (red line), 5 (green line) and 30 nm (blue line) MoS_2 on the top are shown in Fig. S2a. Independently of MoS_2 thickness, the frequency gap between the E^1_{2g} and A_{1g} modes is practically the same. Given that the thickness of 1L MoS_2 is approximately 0.65-0.7 nm [47], the 4L and 8L correspond to 2.6-2.8 nm and 5.1-5.6 nm, respectively. In other words 3 and 5 nm MoS_2 approximately correpond to 4L and 8L MoS_2. However, the gap between E^1_{2g} and A_{1g} modes becomes thickness dependent for MoS_2 with thickness below 4 MoS_2 monolayers [47]. Therefore, the bottom limit for thickness of the studied films of MoS_2 is 4 monolayers.

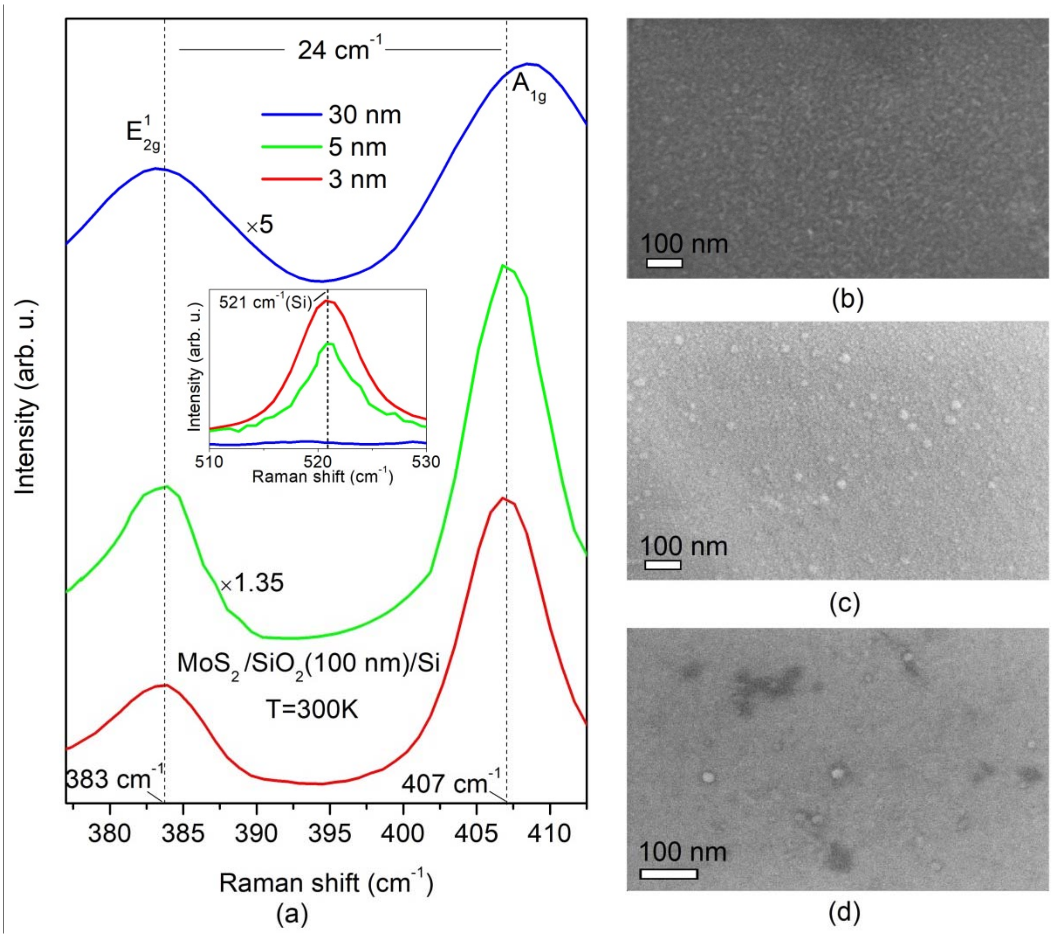

Figure S2. Left panel (a): Raman spectra of the obtained MoS_2/SiO_2 (100 nm)/SiO_2 structures with different thickness of MoS_2 layer (blue, green, and red lines). Inset shows Raman spectra in a wavenumber range above 510 cm^{-1}; Right panel: SEM images of the produced MoS_2 layers with thicknesses of 30 (b), 5 (c), and 3 nm (d).

Therefore, the bottom limit for thickness of the studied films of MoS_2 is 4 monolayers. At the same time, more detailed estimations can also be made from the obtained Raman spectra. For this it is necessary to carefully examine the intensity of the 521 cm^{-1} mode of Si in Raman spectra for each thickness of MoS_2. As shown in the inset in Fig. S2a, this intensity for 5 nm MoS_2 in $MoS_2/SiO_2/Si$ structure is approximately 70% of the intensity achieved in the case of 3 nm thick MoS_2. For 30 nm MoS_2, the 521 cm^{-1} mode is no longer seen in Raman spectra. Simple order-of-magnitude estimations accounting for such intensity behavior of the latter mode of Si with thickness of MoS_2 can be made using values of the absorption coefficient α (Fig. S3) retrieved from the obtained dielectric function for each thickness of MoS_2.

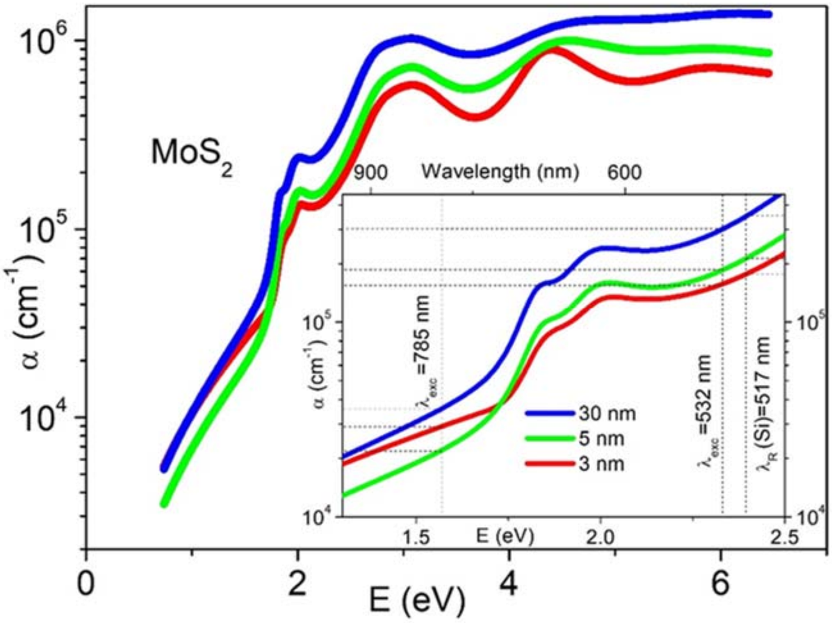

Figure S3. Absorption coefficient as a function of the photon energy for 30 (blue), 5 (green) and 3 nm (red) MoS_2 thin films. Inset shows the details of the photon energy dependence of the absorption coefficient, together with the parameters (excitation wavelength, photon energy) of the lasers used in the present work. Further explanation is given in the text.

Disregarding losses in SiO_2, the ratio (A) of the excitation intensity (I) at the interface between SiO_2 and Si in 5 or 30 nm MoS_2 to the intensity I in 3 nm MoS_2 is easily found from Beer - Lambert law ($I=I_0exp(-\alpha d)$) by using α values at the excitation wavelength of 532 nm (Fig. S3, inset) for each thickness (d) of MoS_2 . The ratio (B) of the intensity of the 521 cm^{-1} mode in 5 or 30 nm MoS_2 to the intensity of this mode in 3 nm MoS_2 is found in the same manner by using data at 517 nm (Fig. S3, inset). Assuming direct proportionality between the intensity of excitation and output Raman intensity and calculating product AxB we find that the intensity of the 521 cm^{-1} mode in 5 nm MoS_2 is less than 0.9 the intensity of this mode in 3 nm MoS_2. For 30 nm MoS_2 the corresponding ratio is approximately 0.1. Both estimates agree fairy well with the experimental data on intensity of the 521 cm^{-1} mode of Si at different thicknesses of MoS_2, underlying mutual consistence of the different physical parameters obtained in the present work. It shall be stressed that by all physical accounts, 30 nm (or 40 L) MoS_2 must be considered as a bulk material.

S3

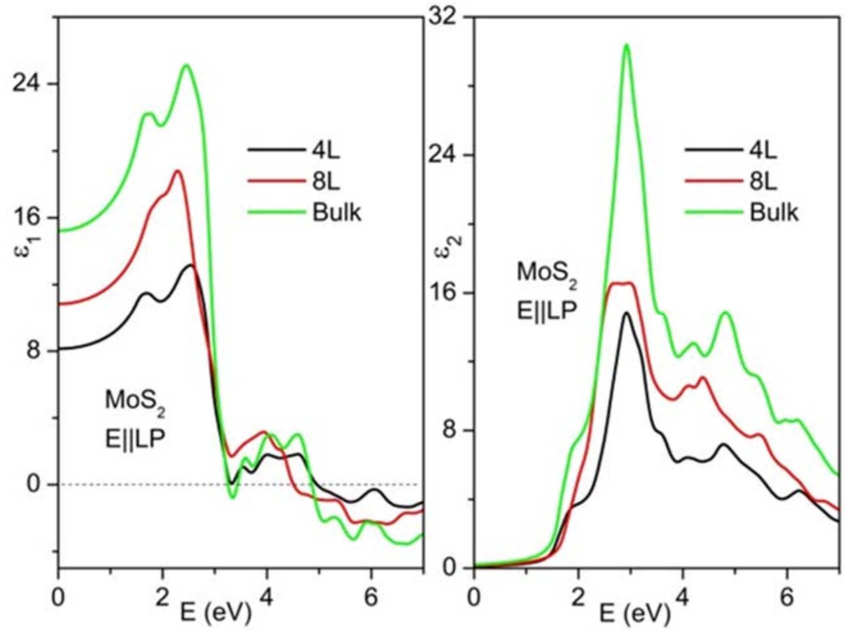

Figure S4. Calculated dielectric function of 4L(black lines), 8L(red lines) and bulk (green lines) MoS_2 for light polarized in the layer plane.

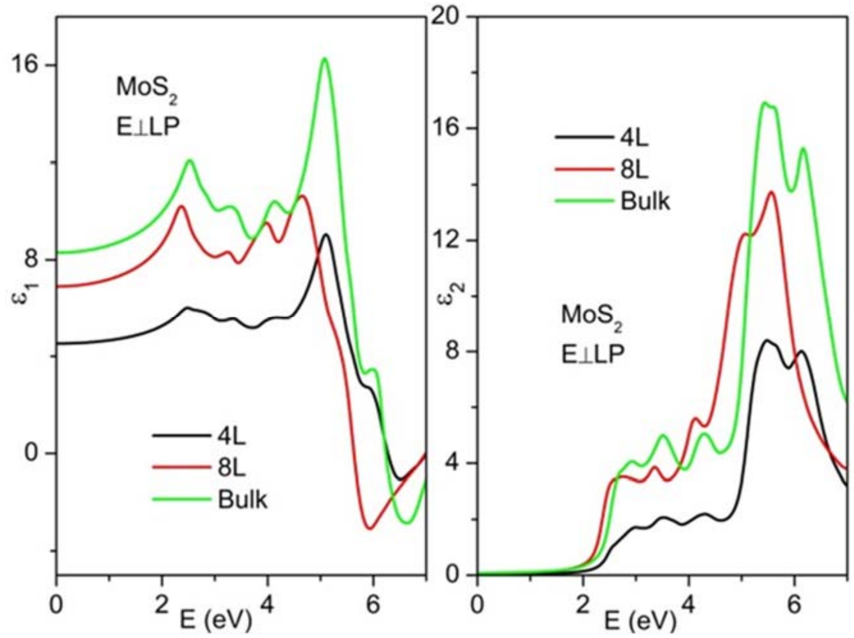

Figure S5. Calculated dielectric function of 4L(black lines), 8L(red lines) and bulk (green lines) MoS_2 for light polarized perpendicular to the layer plane.

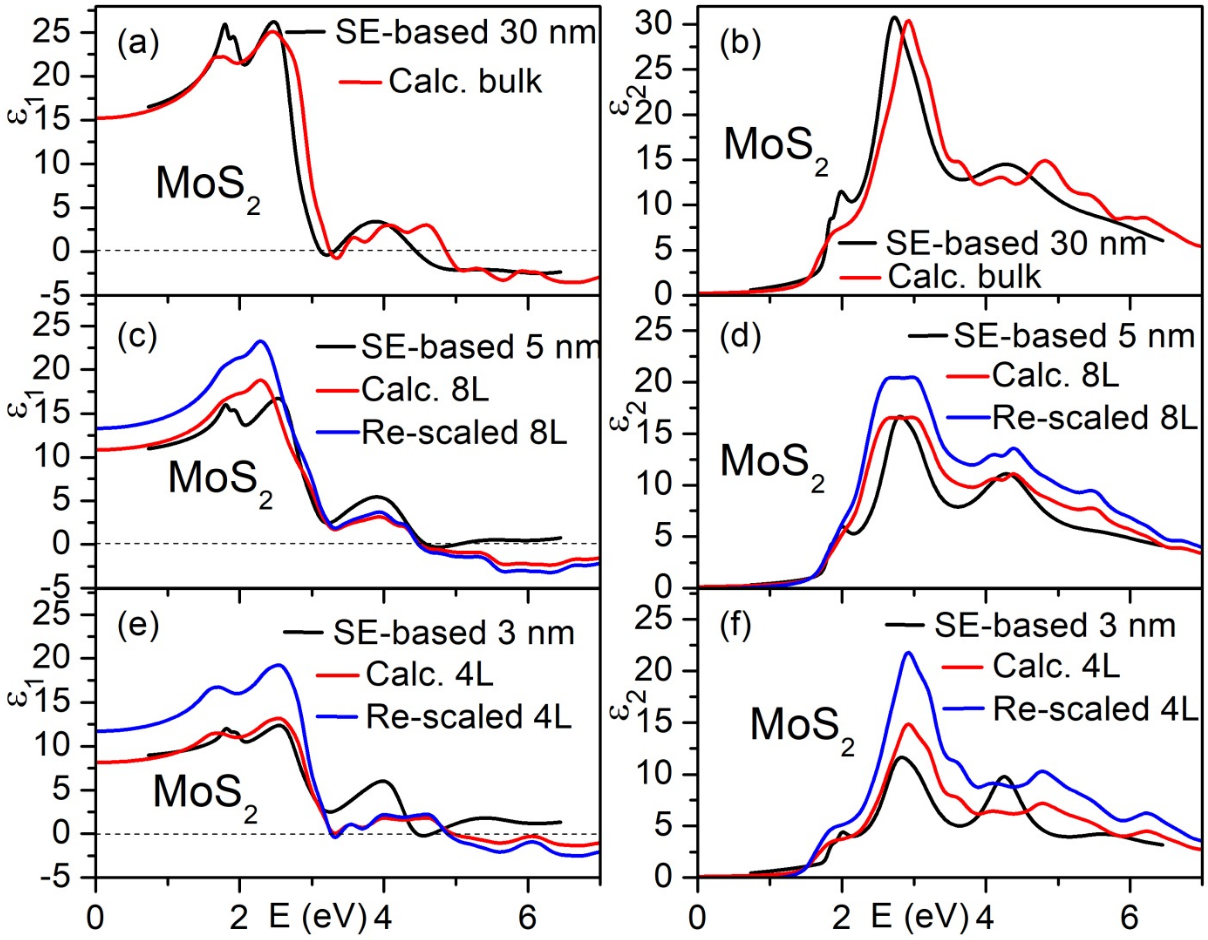

Figure S6. Real and imaginary parts of the experimental (black lines) and calculated (red lines) DF obtained for 30 (a and b) 5 (c and d), and 3 nm (e and f) MoS_2 for light with electrical vector E polarized in the layer plane (LP). The blue curves represent a correction to the calculated DF in order to take into account effects related to the finite number of layers in 5 and 3 nm MoS_2.

S4

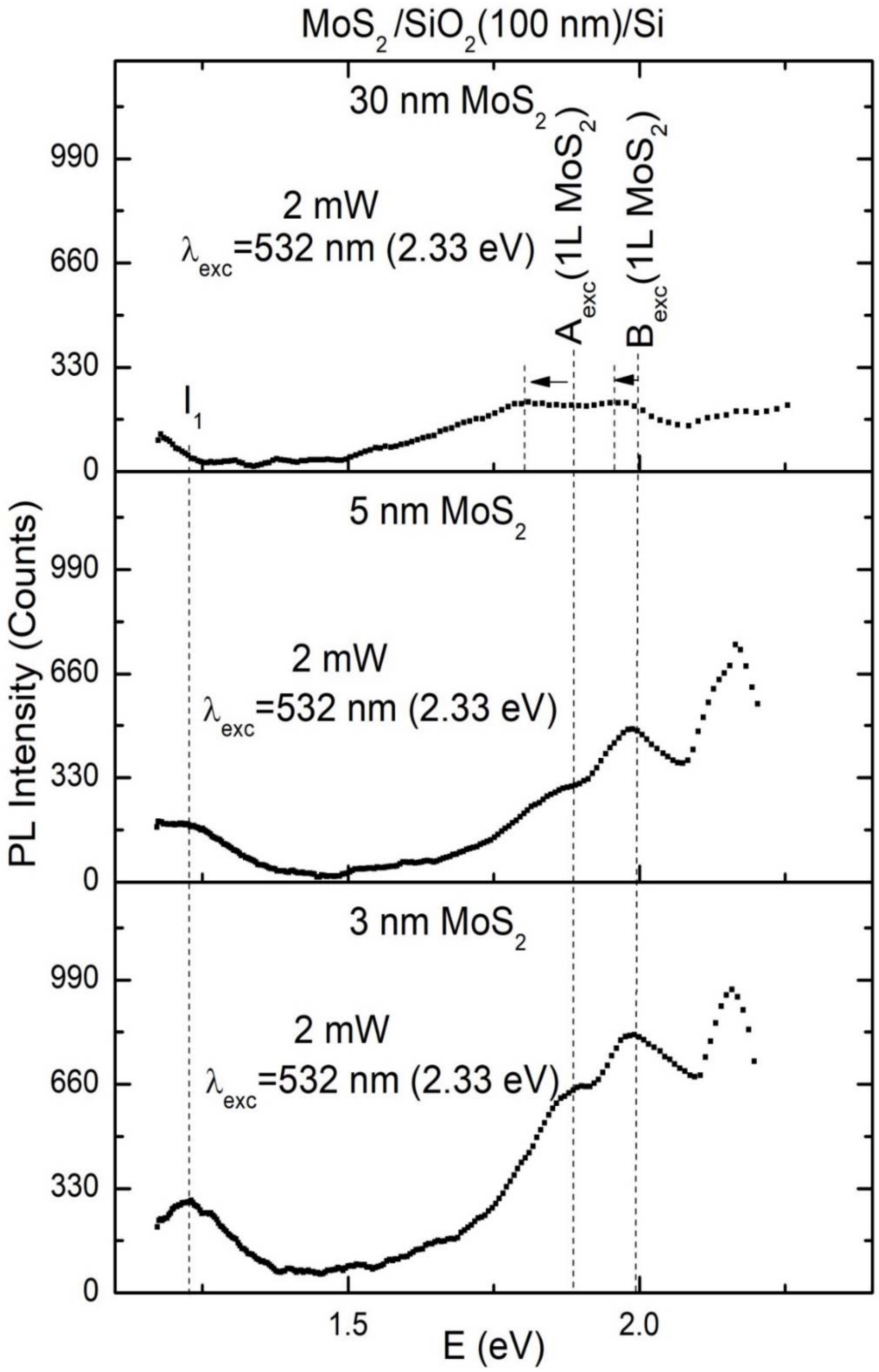

Figure S7. PL spectra of 30 (top part), 5 (middle part) and 30 nm (bottom part) MoS_2. Vertical dashed lines are given for convenience and indicate positions of the indirect transitions I1, A and B excitons in one monolayer of MoS_2 and shift of the A and B exciton positions in 30 nm MoS_2 as compared to 5 and 3 nm MoS_2.

If we compare the difference in exciton positions in PL (Fig. S7) and DF (Fig. 2a) spectra between the 30, 5 and 3 nm MoS_2, it will not be the same for PL and DF. Priority shall be given to DF spectra as they are generally less influenced by the defects and other imperfections.

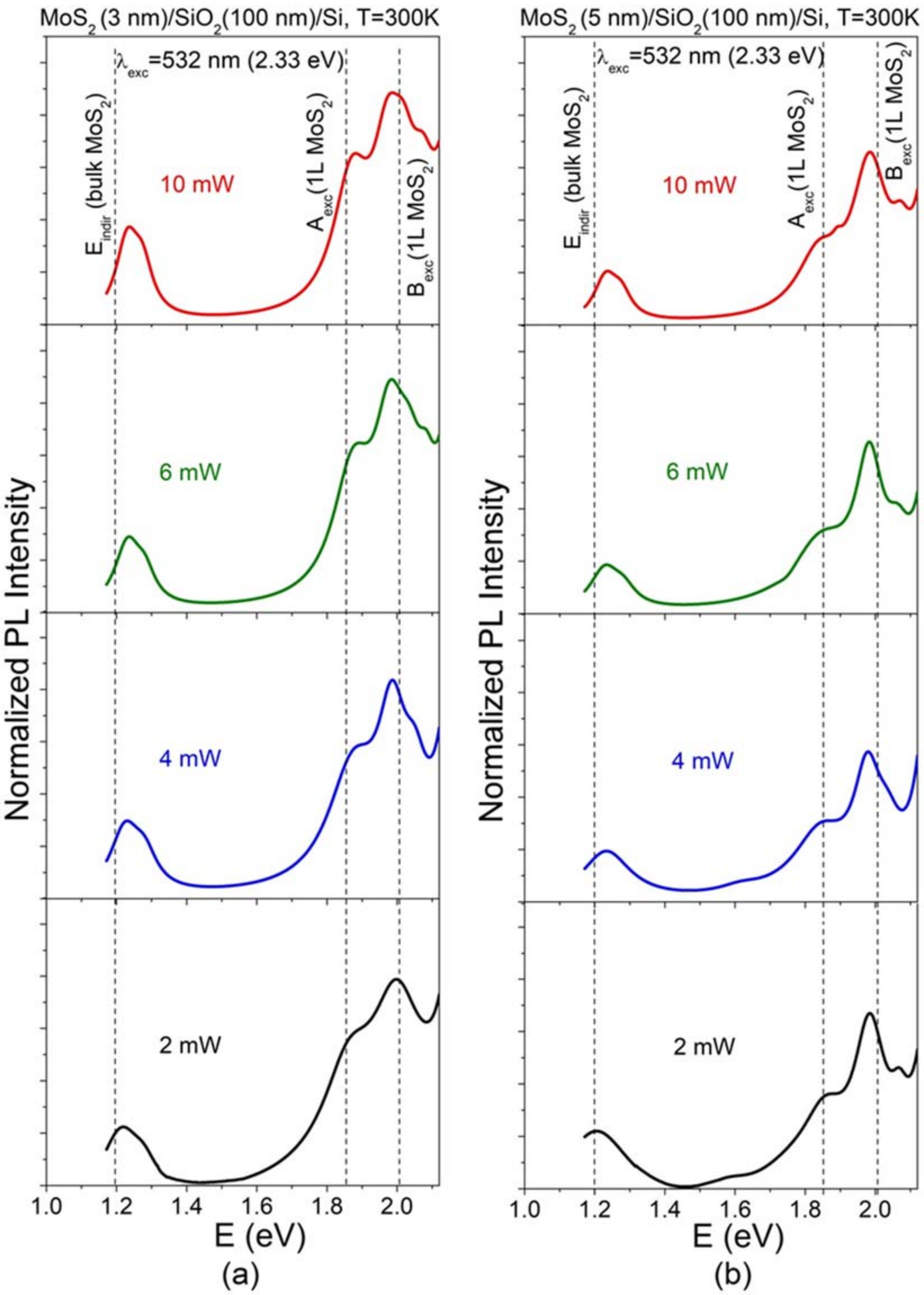

Figure S8. Normalized spectra (line-shape function) of the PL spectra in Figure 3 (main text) ; (a) MoS_2(3 nm)/SiO_2(100 nm)/Si thin film, (b) MoS_2(5 nm)/SiO_2(100 nm)/Si thin film. Two vertical dashed lines on the right and one on the left in each figure are given for comvenience and indicate the positions of excitons (A and B) in monolayer and indirect gap energy in bulk of MoS_2, respectively.

Supplementary Materials References

44. Ferlauto, A.S.; Ferreira, G.M.; Pearce, J.M.; Wronski, C.R.; and Collins, R.W. Analytical model for the optical functions of amorphous semiconductors from the near – infrared to ultraviolet: Applications in thin film Photovoltaics. *J. Appl. Phys.* **2002**, *92 (5)*, 2424 -2436.
45. Mohamed, S.H.; Kappertz, O.; Ngaruiya, J.M.; Leervad-Pedersen, T.P.; Drese, R.; Wuttig, M. Correlation between structure, stress and optical properties in direct current sputtered molybdenum oxide films. *TSF* **2003**, *429*, 135–143.
46. Oiwake, K.; Nishigaki, Y.; Shohei Fujimoto, Sh.; Maeda, S.; and Fujiwara, H. Fully automated spectroscopic ellipsometry analyses: Application to MoOx thin films. *J. Appl. Phys.* **2021**, *129*, 243102 (1-10).
47. Lee, Ch.; Yan, H.; Brus, L.E.; Heinz, T.F.; Hone, J.; and Ryu, S. Anomalous lattice vibrations of single and few-layer MoS_2. *ACS Nano* **2010**, *4(5)*, 2695–2700.
